# The Infant KIdney Dialysis and Utrafiltration (I-KID) Study: A Stepped-Wedge Cluster-Randomized Study in Infants, Comparing Peritoneal Dialysis, Continuous Venovenous Hemofiltration, and Newcastle Infant Dialysis Ultrafiltration System, a Novel Infant Hemodialysis Device

**DOI:** 10.1097/PCC.0000000000003220

**Published:** 2023-03-09

**Authors:** Heather Lambert, Shaun Hiu, Malcolm G. Coulthard, John N. S. Matthews, Eva-Maria Holstein, Jean Crosier, Rachel Agbeko, Thomas Brick, Heather Duncan, David Grant, Quen Mok, Andrew Gustaf Nyman, John Pappachan, Chris Boucher, Joe Bulmer, Denise Chisholm, Kirsten Cromie, Victoria Emmet, Richard G. Feltbower, Arunoday Ghose, Michael Grayling, Rebecca Harrison, Ciara A. Kennedy, Elaine McColl, Kevin Morris, Lee Norman, Julie Office, Roger Parslow, Christine Pattinson, Shriya Sharma, Jonathan Smith, Alison Steel, Rachel Steel, Jayne Straker, Lamprini Vrana, Jenn Walker, Paul Wellman, Mike Whitaker, Jim Wightman, Nina Wilson, Lucy Wirz, Ruth Wood

**Affiliations:** 1 Paediatric Nephrology Department, Great North Children’s Hospital, Royal Victoria Infirmary, The Newcastle Upon Tyne Hospitals NHS Foundation Trust, Newcastle Upon Tyne, United Kingdom.; 2 Biostatistics Research Group, Population Health Sciences Institute, Newcastle University, Newcastle Upon Tyne, United Kingdom.; 3 School of Mathematics, Statistics & Physics, Newcastle University, Newcastle Upon Tyne, United Kingdom.; 4 Newcastle Clinical Trials Unit, Newcastle University, Newcastle Upon Tyne, United Kingdom.; 5 Cardiac Intensive Care Unit, Great Ormond Street Hospital NHS Trust, London, United Kingdom.; 6 Department of Paediatric Intensive Care, Birmingham Women’s and Children’s Hospital, Birmingham, United Kingdom.; 7 Paediatric Intensive Care Unit, Bristol Royal Hospital for Children and University of Bristol Medical School, Bristol, United Kingdom.; 8 Paediatric Intensive Care Unit, Great Ormond Street Hospital for Children, London, United Kingdom.; 9 Paediatric Intensive Care Unit, Evelina London Children’s Hospital, London, United Kingdom.; 10 Paediatric Intensive Care Unit, Southampton Children’s Hospital, Southampton NIHR Biomedical Centre, Southampton, United Kingdom.; 11 Parent, Antrim, Northern Ireland, United Kingdom.; 12 Northern Medical Physics and Clinical Engineering, Royal Victoria Infirmary, Newcastle Upon Tyne, United Kingdom.; 13 Leeds Institute for Data Analytics, School of Medicine, Leeds, United Kingdom.; 14 Clinical Resource Building, Royal Victoria Infirmary, Newcastle Upon Tyne, United Kingdom.; 15 Population Health Sciences Institute, Newcastle University, Newcastle Upon Tyne, United Kingdom.; 16 Institute of Applied Health Research, University of Birmingham, Birmingham, United Kingdom.; 17 Leeds Institute of Cardiovascular and Metabolic Medicine, School of Medicine, Leeds, United Kingdom.; 18 Paediatric Intensive Care Unit, Freeman Hospital, Newcastle Upon Tyne NHS Foundation Trust, Newcastle Upon Tyne, United Kingdom.

**Keywords:** acute kidney injury, dialysis, infant, renal failure, renal replacement therapy, ultrafiltration

## Abstract

Renal replacement therapy (RRT) options are limited for small babies because of lack of available technology. We investigated the precision of ultrafiltration, biochemical clearances, clinical efficacy, outcomes, and safety profile for a novel non-Conformité Européenne-marked hemodialysis device for babies under 8 kg, the Newcastle Infant Dialysis Ultrafiltration System (NIDUS), compared with the current options of peritoneal dialysis (PD) or continuous venovenous hemofiltration (CVVH).

**Design:**

Nonblinded cluster-randomized cross-sectional stepped-wedge design with four periods, three sequences, and two clusters per sequence.

**Setting:**

Clusters were six U.K. PICUs.

**Patients:**

Babies less than 8 kg requiring RRT for fluid overload or biochemical disturbance.

**Interventions:**

In controls, RRT was delivered by PD or CVVH, and in interventions, NIDUS was used. The primary outcome was precision of ultrafiltration compared with prescription; secondary outcomes included biochemical clearances.

**Measurements and Main Results:**

At closure, 97 participants were recruited from the six PICUs (62 control and 35 intervention). The primary outcome, obtained from 62 control and 21 intervention patients, showed that ultrafiltration with NIDUS was closer to that prescribed than with control: sd controls, 18.75, intervention, 2.95 (mL/hr); adjusted ratio, 0.13; 95% CI, 0.03–0.71; *p* = 0.018. Creatinine clearance was smallest and least variable for PD (mean, sd) = (0.08, 0.03) mL/min/kg, larger for NIDUS (0.46, 0.30), and largest for CVVH (1.20, 0.72). Adverse events were reported in all groups. In this critically ill population with multiple organ failure, mortality was lowest for PD and highest for CVVH, with NIDUS in between.

**Conclusions:**

NIDUS delivers accurate, controllable fluid removal and adequate clearances, indicating that it has important potential alongside other modalities for infant RRT.

**RESEARCH IN CONTEXT**

- Renal replacement therapy in children under 8 kg in PICU presents challenges with obtaining access, fluid measurement accuracy, and circuit volume. Improved technology is required.
- The unreliability of fluid balance control during dialysis or hemofiltration may cause hemodynamic instability in small babies.
- The NIDUS, a new hemodialysis and ultrafiltration device specifically designed for patients 0.8–8 kg, was compared with existing treatments with PD and continuous venovenous hemofiltration (CVVH).

**AT THE BEDSIDE**

- The NIDUS delivered more precision of ultrafiltration than control (CVVH and PD) and chemical clearances better than PD but less effectively than CVVH.
- The NIDUS device is clinically effectively, delivering appropriate blood clearances and accurate, controllable fluid removal, with an appropriate safety profile.
- NIDUS has a potential role in the delivery of RRT to some patients.

Critically unwell babies in PICUs may develop acute renal failure and require management with renal replacement therapy (RRT), which is delivered continuously to minimize instability. Their small size and the current limited technology available (which is not licensed in United Kingdom for babies weighing <8 kg) present specific challenges (1, 2). Difficulties with vascular access, blood flows, fluid balance, hypotension, loss of circuits, and filter clotting have been described (3–6). Technical problems are also described for peritoneal dialysis (PD), including leakage and inability to reliably control fluid loss (7). Mortality and morbidity in PICUs varies and is related to the underlying diagnosis: survival is lower in those babies with fluid overload (2, 8), or needing RRT (9).

We undertook the Infant KIdney Dialysis and Utrafiltration (I-KID) study to determine the clinical efficacy, outcomes, and safety profile of a novel non-Conformité Européenne-marked hemodialysis device for infants under 8 kg, the Newcastle Infant Dialysis Ultrafiltration System (NIDUS) (10, 11), compared with current methods of RRT (PD and continuous venovenous hemofiltration [CVVH]). Note that the NIDUS generates most of its solute clearance by diffusion (dialysis) and only some by convection during hemofiltration to produce the required volume of ultrafiltration (UF).

## MATERIALS AND METHODS

The setting was PICUs in six U.K. hospitals, with experience of performing RRT in babies. Children weighing 800 g to 8 kg requiring RRT for fluid overload or biochemical disturbance were recruited: babies with suspected inborn errors of metabolism leading to hyperammonemia were excluded as they require higher than normal dialysis clearances (10). Informed consent was sought from parents/guardians; prospectively, in most cases, but where RRT was urgent, retrospective consent was sought.

We used a cluster-randomized cross-sectional stepped-wedge (SW) design with four periods and three sequences. The periods were each planned to last for 18 weeks: recruitment started in December 2018, but the trial did not close until August 2021 due to pauses in recruitment, largely related to the COVID-19 pandemic. Two sites (PICUs) were randomized to each of the sequences (details in **eAppendix 1**, http://links.lww.com/PCC/C347). Each site was trained in setting up and using the NIDUS before switching to an intervention period. The design gave all participating centers the opportunity to use conventional RRT and NIDUS during the study. Using an SW design permitted phased training on NIDUS and allowed within-center comparisons to contribute to the treatment estimate. PICU nurses were competency assessed before each site could use the intervention. Senior staff delivered cascade-training within their own unit. Newcastle upon Tyne Hospitals (NuTH) staff provided 24-hour telephone/video nursing and medical backup, and rapid medical engineering support.

We measured UF in the CVVH (Prismaflex [Baxter Healthcare, Deerfield, IL] and Aquarius [Edwards Lifesciences, Newbury, Berkshire, United Kingdom]) and NIDUS devices by timed weighings of fresh and waste fluid bags, and in manual PD by measuring the fluid volumes delivered and removed. Timed waste fluid and blood samples were collected to calculate biochemical clearances. Outcome measures are listed in **Table 1**. More details are available in the I-KID Protocol paper (10) and in the NIHR final report (H. Lambert, personal communication, 2023). A comparison of the extracorporeal circuits is shown in **eTable 1** (http://links.lww.com/PCC/C347).

**TABLE 1.** Outcome Measures

Primary outcome
The first available determination of the precision of fluid removal (ultrafiltration) within 48 hours of the start of RRT
Secondary outcomes
Related to the primary outcome
Mean of all ultrafiltration precision values observed on the patient
Precision of observed versus reported fluid removal (continuous venovenous hemofiltration and Newcastle Infant Dialysis and Ultrafiltration System only)
Biochemical clearances
Clearance rates for creatinine, urea, and phosphate
Clinical outcomes
Survival
Hemodynamic instability (defined as a drop in blood pressure requiring intervention, soon after commencement of RRT) including fluid bolus administration and inotrope use
Number of ventilator-free days during RRT
Completion of intended RRT course
Need for additional vascular or dialysis access
Unplanned change in circuits
Exposure to blood transfusion
Bleeding events
Anticoagulant use
Questionnaire outcomes
Parent/guardian experience
Staff acceptability and usability of device

CVVH = continuous venovenous hemofiltration, RRT = renal replacement therapy.

### Interventions

During the control period, the RRT modality was selected from PD and CVVH according to standard unit practice, and NIDUS was used during the intervention period, unless the responsible clinician decided otherwise. One control infant receiving extracorporeal membrane oxygenation (ECMO) had a hemodialysis filter inserted into that circuit to provide RRT. There was no blinding. The study had ethical approval (Tyne and Wear South Research Ethics Committee, reference: 16/NE/0008).

### Statistical Analysis

We denote by *A* and *X*, respectively, the prescribed and achieved UF rates. The planned primary analysis assumed that *X*-*A* had zero mean and compared log|*X*-*A*| between the NIDUS and controls (two groups), which gives a measure of the ratio of the precision of UF between the groups. The sample size calculation, which assumed a two-sided Type I error rate of 5% and a power of 80%, indicated that 96 patients should be recruited to detect a three-fold increase in precision with NIDUS compared with controls: further details are in eAppendix 1 (http://links.lww.com/PCC/C347) and (10). Preliminary analysis indicated that the assumption that *X*-*A* has zero mean was tenable, so the primary analysis compared log|*X*-*A*| between the treatments using a linear model, which also included fixed period and cluster (PICU) effects. Biochemical clearances were compared using a similar model but with PD and CVVH not combined and with intervention-specific residual variances. This model was also used to compare the observed and reported UF for NIDUS and CVVH. Categorical items were compared using χ^2^ tests and CIs for absolute and relative differences.

## RESULTS

**Figure 1.**
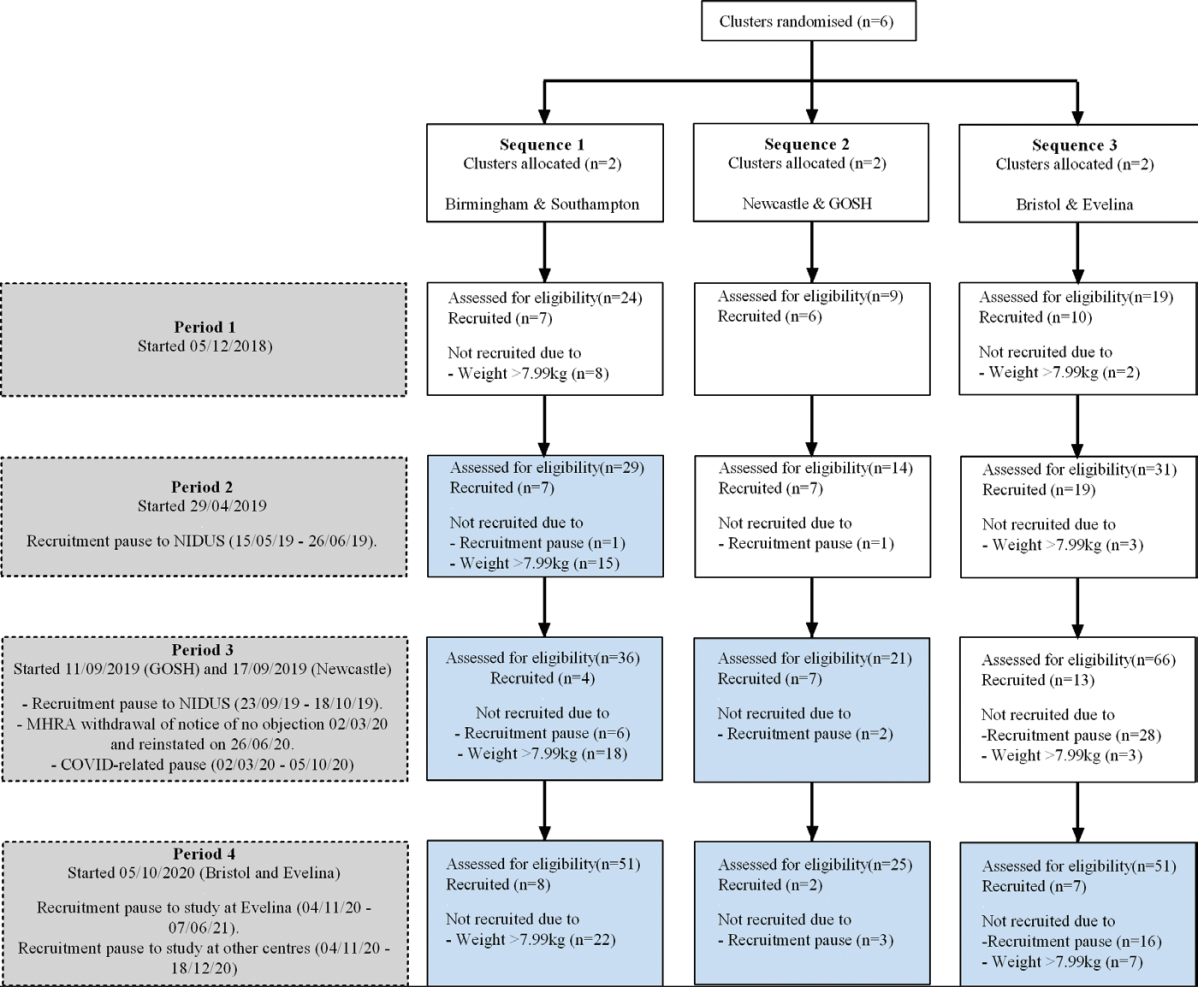
The Consolidated Standards of Reporting Trials patient flow diagram. *Blue boxes* indicate intervention periods. GOSH = Great Ormond Street Hospital, MHRA = Medicines and Healthcare products Regulatory Agency, NIDUS = Newcastle Infant Dialysis Ultrafiltration System.

Ninety-seven patients were recruited, 62 to control, and 35 to intervention (**Fig. 1**), and there were no withdrawals or losses to follow-up. Five recruitment pauses occurred during the study, four due to problems with manufacture of NIDUS consumables, and one because of recruitment restrictions to non-COVID-19 research during the pandemic.

### Baseline Characteristics

The baseline characteristics were similar in control and intervention groups (**eTable 2*A***, http://links.lww.com/PCC/C347); around half the participants had unplanned admissions to PICU, and a third were transferred from other hospitals. RRT was required postsurgery in 52% (32/62) of control and 40% (14/35) of intervention cases. For the participants admitted to PICU postsurgery, this involved the use of cardiopulmonary bypass in 97% (30/31) of controls and 83% (10/12) of intervention participants. Systolic blood pressure median (interquartile range [IQR]) values were control 68 (59–78), and intervention 68 (60–86) mm Hg, and the need for mechanical ventilation was just over 80% in both groups. The median (IQR) age in controls of 10.5 days (7–38 d) was similar to the intervention group 11 days (7–61 d); the overall age range of participants was between 1 and 477 days (c 15 mo). The median (IQR) weights of 3.2 kg (2.9–3.9 kg) and 3.7 kg (3.1–5.6 kg) were similar between control and intervention.

The baseline characteristics by treatment groups (**eTable 2*B***, http://links.lww.com/PCC/C347) appeared balanced with respect to pre-RRT laboratory measurements, though with a slightly higher creatinine and urea values in the intervention arm.

### Precision of Ultrafiltration

**Figure 2.**
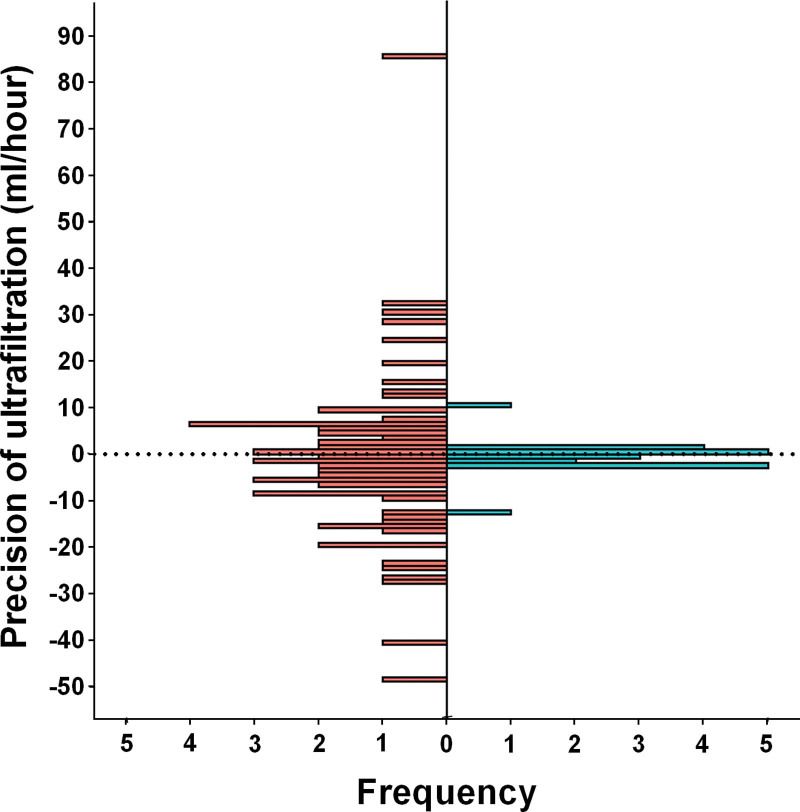
Histogram of ultrafiltration precision, showing the rate achieved versus the rate prescribed. *Red* = control group. *Blue* = intervention group.

UF precision measurements were obtained from all 62 control patients and 21 of the 35 intervention patients. The lack of data for 14 intervention patients was for a variety of reasons, mainly technical difficulties in establishing or sustaining RRT or in measuring UF: see (11) for more details. The variability of the first determination of the achieved UF around the prescribed rate was less with NIDUS compared with control: sds 2.95 vs 18.75 mL/hr, adjusted ratio, 0.13; 95% CI, 0.03–0.71; *p* = 0.018 (**Fig. 2**). If the outcome on a patient is the mean of all available values of log|*X-A*|, the ratio is 0.13; 95% CI, 0.04–0.41.

For NIDUS and CVVH devices, another important measure was to compare the difference between the actual fluid removal rate and that displayed by the device. This had a mean closer to zero for NIDUS than CVVH (means, –0.4 vs 11.6 mL/hr), with less variation in NIDUS than CVVH (sds, 3.2 vs 28.4 mL/hr). See **eTable 3** (http://links.lww.com/PCC/C347) for further descriptive statistics of the primary outcome by PD, CVVH, and NIDUS.

### Biochemical Clearances

**Figure 3.**
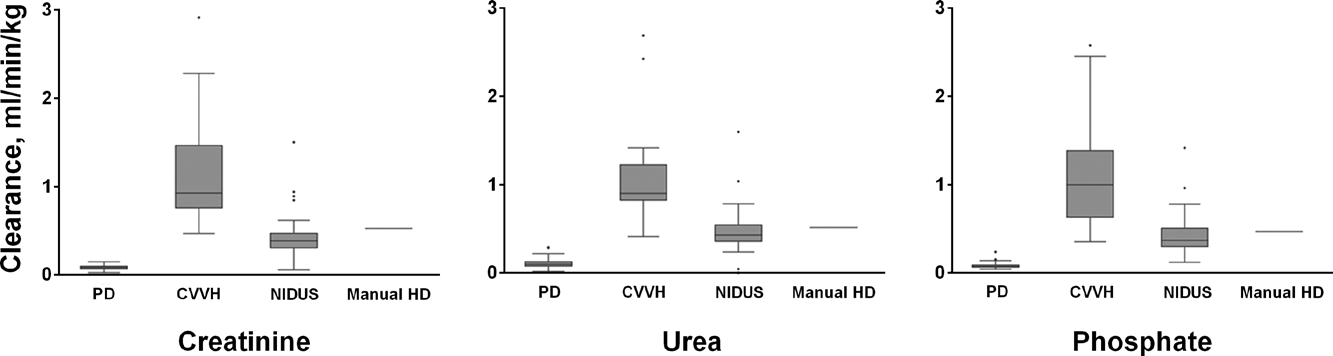
Clearances of creatinine, urea, and phosphate by renal replacement therapy modality. CVVH =continuous venovenous hemofiltration, HD = hemodialysis, NIDUS = Newcastle Infant Dialysis Ultrafiltration System, PD = peritoneal dialysis.

The clearance of creatinine by PD was lower and less variable than by the NIDUS, with mean (sd) values of 0.08 (0.03) vs 0.46 (0.30) mL/min/kg, which was in turn lower and less variable than for CVVH at 1.20 (0.72). This pattern was the same for urea: PD 0.12 (0.06); NIDUS 0.48 (0.30); and CVVH 1.15 (0.67), and for phosphate: PD 0.07 (0.04); NIDUS 0.44 (0.27); and CVVH 1.16 (0.71), all in mL/min/kg (**Fig. 3**). All pairwise treatment comparisons of means and of sds gave *p* < 0.001.

### Outcomes Collected via Paediatric Intensive Care Audit Network

Details of the Paediatric Intensive Care Audit Network outcomes are shown for the control and intervention groups in **eTable 4*A*** (http://links.lww.com/PCC/C347) and for the PD, CVVH, and NIDUS groups in **eTable 4*B*** (http://links.lww.com/PCC/C347).

### Survival

Survival rates until discharge or up to 30-day post start of RRT are in **Table 2** and **eTable 4** (http://links.lww.com/PCC/C347). Nearly all the deaths resulted from congenital abnormalities or sepsis, and the only patient on RRT at the time of death had previously been treated with PD for chronic renal failure (**eTable 5**, http://links.lww.com/PCC/C347).

**TABLE 2.** Survival Data

Treatment Group	30-d Survival		Survival to Discharge	
Control^a^	All = 54/62 (87%)	PD = 47/48 (98%)	All = 52/62 (84%)	PD = 46/48 (96%)
		CVVH = 7/13 (54%)		CVVH = 6/13 (46%)
Newcastle Infant Dialysis and Ultrafiltration System	25/35 (71%)		23/35 (66%)	

CVVH = continuous venovenous hemofiltration, PD = peritoneal dialysis.

a Baby on extracorporeal membrane oxygenation and manual hemodialysis is excluded.

### Venous Access Lines

Six patients (five on CVVH and one on NIDUS) had their venous access lines connected to their ECMO access cannulas. Of the seven CVVH patients that required central venous lines, one required two separate 18-gauge cannulas and six had double-lumen lines. Of the 34 patients treated with NIDUS without ECMO circuits, three used existing 6.5-French central lines, and 31 used single lumens with the following gauges and internal diameters (mm): 16 (1.66) in five; 18 (1.33) in 12; 20 (0.90) in 11; and 22 (0.72) in two.

### Exposure to Blood Transfusion While on RRT

Median (IQR) hemoglobin concentrations prior to starting RRT were similar between the treatment groups. However, babies on PD only received a blood transfusion on a median (quartiles) of 0% (0–0) of the days they were on dialysis (only 15% of these patients received a transfusion at all). This compares with those on NIDUS and CVVH who received transfusions on 43% (8, 67) and 67% (50,75) of days, respectively (eTable 4*B*, http://links.lww.com/PCC/C347). Four of the seven babies treated with CVVH that had their blood accessed directly from their central venous access lines required their 96 mL extracorporeal circuits to be primed with blood rather than saline, but none of those on less than 10-mL NIDUS circuits required blood priming.

### Inotrope Use

The percentage of days a patient was on dialysis when inotropes were given had median (quartiles) 100% (100,100) on PD, 70% (0,100) on CVVH, and 100% (70,100) on NIDUS.

### Fluid Bolus Administration

The fluid volume threshold defined by the PICANet database is greater than or equal to 80 mL/kg. No babies on PD received a bolus this large, four babies did on CVVH, and two did on NIDUS (eTable 4*B*, http://links.lww.com/PCC/C347).

### Safety Reporting

There were seven nonserious adverse events (AEs) in eight control participants and the one nonserious adverse device event (ADE) in an intervention participant (**eTable 6, *A*** and ***B***, http://links.lww.com/PCC/C347). There were 17 serious AEs in 15 participants (eight control and seven intervention), and one serious ADE was reported (**eTable 6, *C*** and ***D***, http://links.lww.com/PCC/C347).

### Questionnaires

Thirty-four parents/care givers of the 97 children described their experience of RRT (four CCVH (two Prismaflex and two Aquarius), 15 PD, and 15 NIDUS. Their responses were generally very positive and highlighted that the study information increased their understanding about their baby’s treatment. Most respondents found it acceptable to be asked to take part in a research study about pediatric RRT despite their child being so unwell and would be likely to recommend future parents to take part in similar research.

Sixty-five staff questionnaires described the experience of delivering RRT to 43 of the 97 trial participants (18 PD, five CVVH [Aquarius], and 20 NIDUS)—with up to five responses, mostly nurses, per patient. There were no identified trends in responses, but most staff felt they had been adequately trained and felt confident using the particular dialysis delivery system. All systems used were considered equally user friendly.

## DISCUSSION

Our data showed that the UF rate the NIDUS delivered was closer to the prescribed value than for CVVH or PD. It also suggested that the achieved UF displayed by the NIDUS was a more reliable reflection of the true UF than was the case for the CVVH devices, although this subgroup analysis may have been potentially influenced by some outliers. This is important because even relatively small imprecisions of UF control may cause relative intravascular hypovolemia in infants; nearly a quarter of infants of 2.5 to 5 kg treated with the CARPEDIEM, a conventional CVVH device which has been miniaturized especially for small body size, required volume replacement for hemodynamic instability (12). Our data also confirm that while it is easy to accurately measure the UF obtained by PD, it is frequently difficult to predict or control.

These UF results are in concordance with previous clinical compassionate use of NIDUS, as well as animal and in vitro testing (11, 13). The higher biochemical clearances of NIDUS compared with PD also reflect previous findings (11), but this is the first comparison between CVVH (Prismaflex and Aquarius) and NIDUS. Given the greater blood flow and larger filter surface area of the CVVH devices, these results are as anticipated. However, the clearances provided by the NIDUS are similar to newborn renal function and, in clinical practice, would provide adequate biochemical control to babies of up to 8 kg with acute renal failure.

The precision of UF and the rates of biochemical clearance support the view that NIDUS is worth pursuing through regulatory procedures as it has a potential place alongside established RRT modalities, particularly for the smallest of infants, which were the original focus for its development.

The survival data reflect the high mortality associated with the underlying clinical diagnoses, but its interpretation is complicated by potential differences in the patients receiving RRT by the different modalities. Although overall mortality was higher in the intervention group, estimates of mortality were smallest for PD and largest for CVVH, with NIDUS in between. These data raise questions about the relative thresholds for starting PD compared with CVVH and NIDUS, and the distribution of complex and unwell babies between these groups. The Pediatric Index of Mortality 3 (PIM3) scores (PIM scoring system based on data collected on admission to PICU) in these groups had medians (quartiles) of 0.02 (0.01–0.05) (PD), 0.027 (0.014–0.131) (NIDUS), and 0.06 (0.01–0.20) (CVVH), although the study was not able to draw any firm conclusions from these statistics (eTable 2*A*, http://links.lww.com/PCC/C347). Some individual clinicians did not enter patients into the I-KID study during the intervention phase if, in their view, clinical considerations dictated otherwise. Thirty-seven of the 45 cases eligible but not recruited for this reason were in period 4, which was mostly postreopening following the COVID-19 moratoria on recruitment, so may reflect other pressures on PICUs.

Babies who are unwell or postsurgical may require blood transfusions for several reasons. Few babies on PD required blood transfusion, but rates were much higher in babies treated with CVVH and NIDUS, which may be partly related to increased blood sampling. We recorded the event and not the volume of blood transfusion. The high rate of transfusion required in the NIDUS group was unexpected compared with clinical experience of NIDUS in the previous pilot study (12) and compassionate use. Patients recruited to I-KID study may have been more unwell, as recruitment was limited to those in PICUs, most of whom had multiple organ failure, compared with those in previous reports not all of whom were in PICU. Half of the CVVH circuits connected to the babies’ central venous lines required blood priming, but none of the NIDUS circuits did.

NIDUS has an acceptable safety profile compared with other modalities used in this critically unwell population. There were AEs reported in both control subgroups and in intervention cases. There were problems related to the NIDUS disposable components and filters, which were addressed and rectified by the manufacturers to complete the study. In the NIDUS group, 60% required an unanticipated filter change compared with 43% on CVVH. These data support the need to redesign the geometry of the filter to reduce its flow resistance while maintaining its membrane surface area and priming volume.

PD is likely to remain a commonly used method for babies with less severe renal failure who require less intensive dialysis. Many postoperative babies (especially those undergoing cardiac surgery) have a PD catheter inserted during surgery, which is sometimes just used for draining ascitic fluid and can be easily used for dialysis as required. However, insertion of a PD catheter is not without its risks, and there is need for future studies questioning the best immediate postoperative renal support modality.

The I-KID study had high input from public and parents at all stages, from the early development phase onward, and this has been crucial in ensuring acceptability to participant parents. Importantly, most parents who responded to our questionnaire indicated they felt it was acceptable to be approached about taking part in research despite the circumstances. This is important for future research studies in critical care. User feedback on NIDUS from I-KID has provided vital information on problems encountered and usability, which require addressing.

### Strengths of the Study

As the first direct comparison between these three different dialysis modalities in infants in PICU, I-KID provides important new information about RRT in babies on PICU. The high input from public and parents at all stages has been crucial, and the study required and achieved a high degree of enthusiasm and support from clinicians and nursing staff. An important safety profile has been created, and I-KID has provided vital information on improvements required to the NIDUS device to improve usability.

### Limitations of Study

The numbers were small; recruitment was high in the first part of the study, when most patients were entering the control phase, but diminished as the study progressed when sites were enrolling babies into the intervention phase. The study faced several challenges to delivery: three pauses due to technical problems with consumables that required resolution by the manufacturers and then the moratoria on non-COVID-19 research lasting between 6 and 16 months (depending on the site) during the COVID pandemic. Even more difficult to quantify was the effect of reopening a study when PICU staff were described as exhausted, and units faced severe staffing shortages.

There were missing and unobtainable data, especially for the primary outcome in the intervention group. We underestimated the effect of staff having to learn a new technology and do additional study tasks at the bedside; study financial provision of additional nursing did not translate directly to availability when needed. The number of control cases on PD compared with CVVH was higher than we had expected based on PICANet data. There was an unanticipated need for additional circuits and frequent filter changes due to filter “sludging” (not clotting), which particularly seemed to affect very sick babies. This prevented completion of some studies. These observations add vital information to support our proposed redesign of the filter housing to reduce baseline-operating pressure. This may also have affected willingness to use of NIDUS in some situations. It should also be remembered that the primary outcome is a measure of the performance of the RRT modality—how well it delivers the prescribed UF rate—and is not closely related to the condition of the patient. Consequently, we believe that the failure to collect UF data from 14 patients in the intervention group has not led to important bias.

Many babies requiring RRT in PICUs are critically unwell, as reflected by the vast majority in I-KID having multiple organ failure; most were on positive pressure ventilatory support. There was a very high use of inotrope infusions, and this may reflect a tendency for “routine use” in babies post cardiac surgery. In retrospect, we did not collect sufficient data on indications for use nor of the details of fluid bolus administration, or of the volumes of blood transfusions.

## CONCLUSIONS

This is the first randomized controlled trial comparing different modalities of RRT completed in infants; our learning from previous experience of a standard crossover trial in NIDUS led to the use of SW design to address this. We had engagement and support from parents and staff due to the perceived need for this device.

Largely, the results were in concordance with clinical experience of RRT in babies and with previous NIDUS animal and compassionate-use evidence (11). Where PD is contraindicated or after PD treatment failure, the NIDUS provides a therapeutic alternative. The results show that the intervention device, NIDUS, works effectively, delivering appropriate blood clearances and accurate, controllable fluid removal, with an appropriate safety profile, indicating that it has an important place alongside other dialysis modalities in the management of babies with renal failure.

## ACKNOWLEDGMENTS

We thank the parents and families of babies recruited to the study for their important contribution, particularly at such a stressful and anxious time when their baby is unwell. We also thank parents and families who advised at various stages in the study for their invaluable contribution. We thank for the work, time, and enthusiasm of the site research and clinical staff in making this study happen, their continuous support, and for enabling restart to recruitment following interruption due to the COVID pandemic. In particular, The Birmingham Women’s & Children’s research team Samantha Owen, Ceri Robbins, Margaret Farley & Helen Winmill; Dr. Michael Griksaitis, Consultant Pediatric Intensivist at Southampton Children’s Hospital; The Great Ormond Street Hospital for Children’s research team Lauran O’Neill, Ana Luisa Tomas, Holly Belfield, Helen Vander-Johnson; The Bristol Royal Hospital for Children’s research team Helen Marley and Becca Lean; and the CVVH clinical teams and research nurses in all of the participating hospitals. We thank the commitment, work, and dedication of the Trial Steering Committee, Trial Management Group, and Independent Data Monitoring Committee as well as the important input from the Sponsor Team at Newcastle upon Tyne Hospitals, in particular Chris Price (Regulatory Compliance Specialist). We thank for the long standing and considerable contribution of members of the Northern Medical Physics and Clinical Engineering Department to the Newcastle Infant Dialysis Ultrafiltration System (NIDUS) project, including Alison Bray and Michael Drinnan. We thank Allmed for loan of NIDUS devices.

## Supplementary Material


