# Supplementary material for: The Infant KIdney Dialysis and Utrafiltration (I-KID) Study: A Stepped-Wedge Cluster-Randomized Study in Infants, Comparing Peritoneal Dialysis, Continuous Venovenous Hemofiltration, and Newcastle Infant Dialysis Ultrafiltration System, a Novel Infant Hemodialysis Device
Source: Pediatr Crit Care Med. 2023 Mar 9;24(7):604–13. doi: 10.1097/PCC.0000000000003220 (PMC10317301; doi:10.1097/PCC.0000000000003220)
Supplement: Supplementary file 1 [file pcc-24-604-s001.docx]

**Supplemental digital content for the I-KID study**

**Contents**

| **Item** | **Title** | **Page** |
| --- | --- | --- |
| eAppendix 1 | The stepped wedge design in I-KID | 2 |
| eTable 1 | Details of filters and circuit volumes | 4 |
| eTable 2a | Baseline characteristics by control or intervention | 5 |
| eTable 2b | Baseline characteristics by RRT modality | 8 |
| eTable 3 | Descriptive statistics of the primary outcome by PD, CVVH, and NIDUS | 11 |
| eTable 4a | Descriptive and inferential statistics of PICANet outcomes by control and intervention groups | 12 |
| eTable 4b | Descriptive statistics of PICANet outcomes PD, CVVH and NIDUS | 14 |
| eTable 5 | Causes of death and relationship with devices | 16 |
| eTable 6a | Non-serious adverse events (AEs) which were not consistent with the usual clinical pattern for participants requiring RRT in PICU | 18 |
| eTable 6b | Adverse device events (ADEs, only collected in intervention for NIDUS). | 19 |
| eTable 6c | Serious adverse events (SAEs) | 20 |
| eTable 6d | Serious adverse device event (SADE) | 22 |

**Appendix 1 The Stepped Wedge design in I-KID**

|  | Period 1 | Period 2 | Period 3 | Period 4 |
| --- | --- | --- | --- | --- |
| Sequence 1 | Control | NIDUS | NIDUS | NIDUS |
| Sequence 2 | Control | Control | NIDUS | NIDUS |
| Sequence 3 | Control | Control | Control | NIDUS |

**Choice of Design**

A cluster-randomised Stepped Wedge (SW) design was chosen for I-KID for the following reasons.

1. Randomising PICUs (clusters) rather than patients meant that at any time during the trial, all patients in a given PICU would be receiving the same treatment. This was deemed preferable for both practical and ethical reasons.
2. The SW variant of a cluster-randomised allowed the use of NIDUS to be phased in over the duration of the trial, which facilitated the training staff at each site needed before using the new method.
3. The treatment estimate from a SW design aggregates across PICUs comparisons that have been made between patients in the same cluster, thereby reducing the impact of inter-unit differences in practice.

**Sample size calculation**

In addition to the details given in the paper, the sample size calculation used historical data to provide the expected annual recruitment of eligible patients at each PICU (taken as 14, 14, 14, 9, 9 and 3) and used this information to determine that each period should last 18 weeks. It was assumed that the *X*-*A* had zero mean and followed a Normal distribution, so the standard deviation of log|*X*-*A*| was π/(2√2)≈1.11. Initial planning assumed an intra-class correlation of 0.1, based on a simple compound symmetry correlation structure. This assumption became questionable following changes to the intervals between periods because of pauses to recruitment due to the Covid-19 pandemic. Consequently the final analysis used fixed cluster effects as this made fewer assumptions (see Matthews JNS, Forbes AB, Stepped wedge designs: insights from a design of experiments perspective, *Statistics in Medicine*, 2017, 36, 3772-3790). The calculations used the formulae in Matthews & Forbes, adapted to unequal cluster sizes, and implemented using a bespoke R program. Further details are given in the report to the funder.

### Randomisation of the stepped-wedge design

The design for I-KID was a three-sequence SWD and six sites (PICUs), allocating two sites to each sequence. Three sites were expected to recruit at rates about 50% higher than the other three sites: it was convenient to designate the first three sites as large sites and the others as small sites. The randomisation was restricted so that one large site and one small site was allocated to each sequence, to mitigate the risk of a sequence with very low recruitment and to control the variation in power between different allocations.

The procedure for randomisation was as follows. The Senior Trial Manager produced a list in which each of the symbols A, B and C was associated with one of the large sites in an arbitrary order. A second list associated the symbols a, b and c with the small sites. These lists were not revealed to any other member of the TMG. The Senior Trial Statistician used the base function sample in R to produce a random permutation of the symbols A, B and C, with the first element of the permuted list being allocated to sequence 1, the second to sequence 2 and the last to sequence 3. This was repeated for the symbols a, b and c. This allocation, with one upper case and one lower case letter allocated to each sequence was passed to the Senior Trial Manager, who was able to form the random allocation of sites to sequences by substituting the actual site names for the symbols.

The allocations were revealed to the sites in sequence 1 only as far ahead of the end of period 1 as was necessary for training and for the sites to make appropriate practical arrangements. At this stage the allocations for the remaining sites were not revealed. The same procedure was adopted for sites changing over after period 2, although in this case concealment of the allocation of the sites to sequence 3 was unnecessary.

**eTable 1 – Details of the filters and circuit volumes**

| Device | Filter name | | | Circuit |
| --- | --- | --- | --- | --- |
|  | Name | Material | Surface area (m^2^) | Volume (ml) |
| NIDUS | NeoFlux1 | Polysulfone | 0.045 | 9.5 |
| Prismaflex | HF20 | Polysulfone | 0.2 | 60 |
| Aquarius | HF03 | Polysulfone | 0.3 | 96 |

**eTable 2a - Baseline characteristics by control or intervention (page 1)**

|  | **Control (n=62)** | **Intervention (n=35)** | **Total (n=97)** |
| --- | --- | --- | --- |
| Age at screening, days |  |  |  |
| Mean (SD) | 52.1 (100.8) | 91.7 (137.0) | 66.4 (116.0) |
| Median (IQR) | 10.5 (7, 38) | 11 (7, 124) | 11 (7, 61) |
| Range | 1, 477 | 1, 443 | 1, 477 |
| Available n | 62 | 35 | 97 |
| Sex, n (%) |  |  |  |
| Female | 27 (44) | 8 (23) | 35 (36) |
| Male | 35 (56) | 27 (77) | 62 (64) |
| Weight at RRT initiation, kg^a^ |  |  |  |
| Mean (SD) | 3.76 (1.59) | 4.33 (1.72) | 3.97 (1.65) |
| Median (IQR) | 3.20 (2.90, 3.90) | 3.70 (3.10, 5.60) | 3.50 (3.00, 4.60) |
| Range | 1.80, 10.10 | 1.00, 7.80 | 1.00, 10.10 |
| Available n | 62 | 35 | 97 |
| Type of weight measurement, n  (%) |  |  |  |
| Actual weight | 51 (82) | 34 (97) | 85 (88) |
| Estimated weight | 11 (18) | 1 (3) | 12 (12) |
| Gestational age at delivery (completed weeks) |  |  |  |
| Mean (SD) | 37.9 (2.3) | 36.5 (3.7) | 37.4 (3.0) |
| Median (IQR) | 38 (37, 39) | 38 (35, 39) | 38 (37, 39) |
| Range | 28, 41 | 26, 41 | 26, 41 |
| Available n | 61 | 35 | 96 |

^a^One participant on Prismaflex® had a total body weight of 10.1 kg but an estimated dry weight of 7.7 kg, and was deemed acceptable for study eligibility

**eTable 2a - Baseline characteristics by control or intervention (page 2)**

|  | **Control (n=62)** | **Intervention (n=35)** | **Total (n=97)** |
| --- | --- | --- | --- |
| Type of admission to unit, n (%) |  |  |  |
| Post-Surgery, Planned | 31 (50) | 13 (37) | 44 (45) |
| Post-Surgery, Unplanned | 1 (2) | 1 (3) | 2 (2) |
| Non-Surgical; Multiorgan failure | 30 (48) | 21 (60) | 51 (53) |
| Previous ICU admission, n (%) |  |  |  |
| ICU | 0 | 1 (3) | 1 (1) |
| PICU | 4 (6) | 4 (11) | 8 (8) |
| NICU | 29 (47) | 12 (34) | 41 (42) |
| None | 26 (42) | 18 (51) | 44 (45) |
| Unknown | 3 () | 0 | 3 (3) |
| Source of admission, n (%) |  |  |  |
| Same hospital | 40 (65) | 23 (66) | 63 (65) |
| Other hospital | 22 (35) | 12 (34) | 34 (35) |
| Elective admission, n (%) |  |  |  |
| No | 29 (47) | 18 (51) | 47 (48) |
| Yes | 33 (53) | 17 (49) | 50 (52) |
| Main reason for PICU admission, n (%) |  |  |  |
| Other | 30 (48) | 20 (57) | 50 (52) |
| Bronchiolitis | 1 (2) | 2 (6) | 3 (3) |
| Recovery from surgery | 31 (50) | 12 (34) | 43 (44) |
| Seizure disorder | 0 | 1 (3) | 1 (1) |
| If admission was recovery from surgery, what was procedure, n (%) |  |  |  |
| Bypass cardiac procedure | 30 (97) | 10 (83) | 40 (93) |
| Non-bypass cardiac procedure | 0 | 1 (8) | 1 (2) |
| Other procedure | 1 (3) | 1 (8) | 2 (5) |

**eTable 2a - Baseline characteristics by control or intervention (page 3)**

|  | **Control (n=62)** | **Intervention (n=35)** | **Total (n=97)** |
| --- | --- | --- | --- |
| Systolic blood pressure, mmHg |  |  |  |
| Mean (SD) | 72.2 (19.5) | 74.9 (22.3) | 73.2 (20.5) |
| Median (IQR) | 68 (59, 78) | 68.00 (60, 86) | 68 (60, 82) |
| Range | 40, 137 | 36, 134 | 36, 137 |
| Available n | 59 | 35 | 94 |
| Mechanical ventilation, n (%) |  |  |  |
| Yes | 50 (81) | 30 (86) | 80 (82) |
| No | 12 (19) | 5 (14) | 17 (18) |
| PIM3 score^b^ |  |  |  |
| Mean (SD) | 0.07 (0.10) | 0.095 (0.172) | 0.079 (0.130) |
| Median (IQR) | 0.02 (0.01, 0.07) | 0.03 (0.01, 0.13) | 0.03 (0.01, 0.09) |
| Range | 0.005, 0.445 | 0.006, 0.972 | 0.005, 0.972 |
| Available n | 62 | 35 | 97 |

^b^ PIM3 score = paediatric index of mortality 3.

**eTable 2b Baseline characteristics by the modality of RRT (page 1)**

|  | **PD (n=48)** | **CVVH (n=13)** | **Manual HD (n=1)** | **NIDUS® (n=35)** | **Total (n=97)** |
| --- | --- | --- | --- | --- | --- |
| Age at screening, days |  |  |  |  |  |
| Mean (SD) | 42.0 (85.9) | 92.6 (142.9) | 11 (.) | 91.66 (136.85) | 66.37 (115.99) |
| Median (IQR) | 9 (6.5, 17.5) | 17 (9, 81) |  | 11 (7, 124) | 11 (7, 61) |
| Range | 1, 477 | 1, 466 |  | 1, 443 | 1, 477 |
| Available n | 48 | 13 | 1 | 35 | 97 |
| Sex, n(%) |  |  |  |  |  |
| Female | 20 (42) | 6 (46) | 1 (100) | 8 (23) | 35 (36) |
| Male | 28 (58) | 7 (54 | 0 | 27 (77) | 62 (64) |
| Weight at RRT initiation, kg* |  |  |  |  |  |
| Mean (SD) | 3.70 (1.29) | 4.25 (2.43) | 2.70 (.) | 4.33 (1.72) | 3.97 (1.65) |
| Median (IQR) | 3.25 (2.90, 3.90) | 3.00 (2.75, 4.00) |  | 3.70 (3.10, 5.60) | 3.50 (3.00, 4.60) |
| Range | 1.80, 7.40 | 2.60, 10.10 |  | 1.00, 7.80 | 1.00, 10.10 |
| Available n | 48 | 13 | 1 | 35 | 97 |
| Type of weight measurement, n  (%) |  |  |  |  |  |
| Actual weight | 40 (83) | 10 (77) | 1 (100) | 34 (97) | 85 (88) |
| Estimated weight | 8 (17) | 3 (23) | 0 | 1 (3) | 12 (12) |
| Gestational age at delivery (completed weeks) |  |  |  |  |  |
| Mean (SD) | 38.1 (1.9) | 37.3 (3.3) | 38 (.) | 36.5 (3.7) | 37.4 (3.0) |
| Median (IQR) | 38 (38, 39) | 37.5 (36.5, 39.5) |  | 38 (35, 39) | 38 (37, 39) |
| Range | 31, 41 | 28, 41 |  | 26, 41 | 26, 41 |
| Available n | 48 | 12 | 1 | 35 | 96 |

^a^One participant on Prismaflex® had a total body weight of 10.1 kg but an estimated dry weight of 7.7 kg, and was deemed acceptable for study eligibility

**eTable 2b Baseline characteristics by the modality of RRT (page 2)**

|  | **PD (n=48)** | **CVVH (n=13)** | **Manual HD (n=1)** | **NIDUS® (n=35)** | **Total (n=97)** |
| --- | --- | --- | --- | --- | --- |
| Type of admission to unit, n (%) |  |  |  |  |  |
| Planned - Following Surgery | 30 (63) | 1 (8) | 0 | 13 (37) | 44 (45) |
| Unplanned - Following Surgery | 1 (2) | 0 | 0 | 1 (3) | 2 (2) |
| Planned - Other | 1 (2) | 1 (8) | 0 | 4 (11) | 6 (6) |
| Unplanned | 16 (33) | 11 (85) | 1 (100) | 17 (49) | 45 (46) |
| Previous ICU admission, n (%) |  |  |  |  |  |
| ICU | 0 | 0 | 0 | 1 (3) | 1 (1) |
| PICU | 3 (6) | 1 (8) | 0 | 4 (11) | 8 (8) |
| NICU | 22 (46) | 7 (54) | 0 | 12 (34) | 41 (42) |
| None | 21 (44) | 4 (31) | 1 (100) | 18 (51) | 44 (45) |
| Unknown | 2 (4) | 1 (8) | 0 | 0 | 3 (3) |
| Source of admission, n (%) |  |  |  |  |  |
| Same hospital | 33 (69) | 6 (46) | 1 (100) | 23 (66) | 63 (65) |
| Other hospital | 15 (31) | 7 (54) | 0 | 12 (34) | 34 (35) |
| Elective admission, n (%) |  |  |  |  |  |
| No | 17 (35) | 11 (85) | 1 (100) | 18 (51) | 47 (48) |
| Yes | 31 (65) | 2 (15) | 0 | 17 (49) | 50 (52) |
| Main reason for PICU admission, n (%) |  |  |  |  |  |
| Other | 18 (38) | 11 (85) | 1 (100) | 20 (57) | 50 (52) |
| Bronchiolitis | 0 | 1 (8) | 0 | 2 (6) | 3 (3) |
| Recovery from surgery | 30 (62) | 1 (8) | 0 | 12 (34) | 43 (44) |
| Seizure disorder | 0 | 0 | 0 | 1 (3) | 1 (1) |

**eTable 2b Baseline characteristics by the modality of RRT (page 3)**

|  | **PD (n=48)** | **CVVH (n=13)** | **Manual HD (n=1)** | **NIDUS® (n=35)** | **Total (n=97)** |
| --- | --- | --- | --- | --- | --- |
| If admission was recovery from surgery, what was procedure, n  (%) |  |  |  |  |  |
| Bypass cardiac procedure | 30 (100) | 0 | 0 | 10 (83) | 40 (93) |
| Non-bypass cardiac procedure | 0 | 1 (100) | 0 | 1 (8) | 1 (2) |
| Other procedure | 0 |  |  | 1 (8) | 2 (5) |
| Is evidence available to assess past medical history, n (%) |  |  |  |  |  |
| Yes | 42 (87) | 12 (92) | 1 (100) | 35 (100) | 90 (93) |
| No | 6 (13) | 1 (8) | 0 | 0 | 7 (7) |
| Systolic blood pressure, mmHg |  |  |  |  |  |
| Mean (SD) | 68.4 (15.2) | 83.1 (27.2) | 100 (.) | 74.9 (22.3) | 73.2 (20.5) |
| Median (IQR) | 66 (57, 74) | 77 (65, 92) |  | 68 (60, 86) | 68 (60, 82) |
| Range | 40, 118 | 44, 137 |  | 36, 134 | 36, 137 |
| Available n | 45 | 13 | 1 | 35 | 94 |
| Mechanical ventilation, n(%) |  |  |  |  |  |
| Yes | 41 (85) | 9 (69) | 1 (100) | 30 (86) | 80 (82) |
| No | 7 (15) | 4 (31) | 0 | 5 (14) | 17 (18) |
| PIM3 score^b^ |  |  |  |  |  |
| Mean (SD) | 0.06 (0.09) | 0.12 (0.13) | 0.02 (.) | 0.10 (0.17) | 0.08 (0.13) |
| Median (IQR) | 0.02 (0.01, 0.05) | 0.06 (0.01, 0.20) |  | 0.03 (0.01, 0.13) | 0.03 (0.01, 0.09) |
| Range | 0.00, 0.45 | 0.01, 0.38 |  | 0.006, 0.972 | 0.005, 0.972 |

^b^ PIM3 score = paediatric index of mortality 3.

eTable 3 - Descriptive statistics of the primary outcome by PD, CVVH, and NIDUS

| Variable | PD (n=48) | CVVH (n=13) | NIDUS (n=20) |
| --- | --- | --- | --- |
| *X*, ml/hr |  |  |  |
| Mean (SD) | 8.13 (7.92) | 38.74 (37.19) | 14.88 (9.37) |
| Median (IQR) | 6.67 (2.75, 12.42) | 31.85 (20.64, 40.00) | 12.88 (9.08, 19.57) |
| Range | -5.17, 27.83 | 0.47, 143.32 | 0.54, 36.67 |
| Available n | 48 | 13 | 20 |
| *A*, ml/hr |  |  |  |
| Mean (SD) | 11.79 (12.57) | 30.56 (15.60) | 15.36 (10.14) |
| Median (IQR) | 10.00 (4.00, 13.50) | 30.22 (20.00, 39.03) | 12.26 (8.58, 21.15) |
| Range | 0.00, 60.00 | 6.23, 58.00 | 0.00, 40.00 |
| Available n | 48 | 13 | 20 |
| Precision (*X*-*A*), ml/hr |  |  |  |
| Mean (SD) | -3.67 (15.10) | 8.19 (27.29) | -0.48 (2.95) |
| Median (IQR) | -1.40 (-10.92, 5.12) | -1.67 (-6.13, 7.24) | -0.55 (-2.48, 0.26) |
| Range | -49.17, 27.83 | -15.65, 85.32 | -3.33, 10.32 |
| Available n | 48 | 13 | 20 |
| log\|*X*-*A*\| |  |  |  |
| Mean (SD) | 1.82 (1.30) | 1.90 (1.80) | -0.02 (1.41) |
| Median (IQR) | 1.88 (1.15, 2.73) | 1.98 (1.59, 2.75) | 0.31 (-0.72, 0.94) |
| Range | -3.00, 3.90 | -3.03, 4.45 | -3.91, 2.33 |
| Available n | 48 | 13 | 20 |

^The patient on manual HD and ECMO was not included. One baby on NIDUS® was the first to be recruited at a site.^

eTable 4a - Descriptive and inferential statistics of PICANet outcomes by control and intervention groups (page 1)

|  | **Control (n=62)** | **Intervention (n=35)** | **Total (n=97)** | **χ^2^, df, p** | **Difference in percentage [95% CI]** | **OR [95% CI]** |
| --- | --- | --- | --- | --- | --- | --- |
| Completion of planned RRT, n (%) |  |  |  |  |  |  |
| No | 4 (8) | 11 (52) | 15 (21) | χ^2^(1)=17.07, p<0.001 | -44  [‑67, ‑22] | 0.08  [0.02, 0.31] |
| Yes | 45 (92) | 10 (48) | 55 (79) |  |  |  |
| Missing | 13 | 14 | 27 |  |  |  |
| Survival until PICU discharge, n (%) |  |  |  |  |  |  |
| Alive | 52 (84) | 23 (66) | 75 (77) | χ^2^(1)=4.21, p=0.040 | 18  [0, 36] | 2.71  [1.03, 7.27] |
| Dead | 10 (16) | 12 (34) | 22 (23) |  |  |  |
| Survival until 30 days follow-up, n  (%) |  |  |  |  |  |  |
| Alive | 54 (87) | 25 (71) | 79 (81) | χ^2^(1)=3.63, p=0.057 | 16  [-1, 33] | 2.70  [0.95, 7.67] |
| Dead | 8 (13) | 10 (29) | 18 (19) |  |  |  |
| Need for additional vascular or dialysis access on RRT whilst in PICU, n (%) |  |  |  |  |  |  |
| Yes | 62 (100.00) | 35 (100.00) | 97 (100.00) |  |  |  |
| Haemodynamic status (drop in blood pressure after connection requiring intervention), n (%) |  |  |  |  |  |  |
| No | 3 (5) | 4 (11) | 7 (7) | χ^2^ (1)=1.41, p=0.228 | -7 [-18, 5] | 0.39 [0.08, 1.87] |
| Yes | 59 (95) | 31 (89) | 90 (93) |  |  |  |

**eTable 4a - Descriptive and inferential statistics of PICANet outcomes by control and intervention groups (page 2)**

| Unplanned filter change on RRT whilst in PICU, n (%) |  |  |  |  |  |  |
| --- | --- | --- | --- | --- | --- | --- |
| No | 55 (89) | 14 (40) | 69 (71) | χ^2^(1)=25.85, p<0.001 | 49  [31, 67] | 11.79  [4.18, 33.25] |
| Yes | 7 (11) | 21 (60) | 28 (29) |  |  |  |
| Exposure to blood transfusion on RRT whilst in PICU, n (%) |  |  |  |  |  |  |
| No | 42 (68) | 8 (23) | 50 (52) | χ^2^(1)=18.05, p<0.001 | 45  [27, 63] | 7.09  [2.74, 18.36] |
| Yes | 20 (32) | 27 (77) | 47 (48) |  |  |  |
| Ventilation-free days on RRT whilst in PICU |  |  |  |  |  |  |
| Mean (SD) | 0.4 (1.6) | 1.7 (8.3) | 0.8 (5.2) |  |  |  |
| Median (IQR) | 0 (0, 0) | 0 (0, 0) | 0 (0, 0) |  |  |  |
| Range | 0, 12 | 0, 49 | 0, 49 |  |  |  |
| Available n | 62 | 35 | 97 |  |  |  |

eTable 4b - Descriptive statistics of PICANet outcomes by PD, CVVH, and NIDUS (page 1)

|  | PD (n=48) | CVVH (n=13) | NIDUS (n=35) |
| --- | --- | --- | --- |
| Completion of planned RRT, n (%) |  |  |  |
| No | 3 (6) | 1 (8) | 11 (31) |
| Yes | 41 (85) | 4 (31) | 10 (29) |
| Missing | 4 (8) | 8 (62) | 14 (40) |
| Need for additional vascular or dialysis access on RRT whilst in PICU, n (%) |  |  |  |
| Yes | 48 (100) | 13 (100) | 35 (100) |
| Haemodynamic status (drop in blood pressure after connection requiring intervention),  n (%) |  |  |  |
| No | 0 | 3 (23) | 4 (11) |
| Yes | 48 (100) | 10 (77) | 31 (89) |
| Fluid bolus given **^a^** |  |  |  |
| No | 48 (100) | 9 (69) | 33 (94) |
| Yes | 0 | 4 (31) | 2 (6) |
| Summary statistics ^b^ | 0.0,0.0,0.0,0.0,0.0 | 0.0,0.0,0.0,0.1,1.0 | 0.0,0.0,0.0,0.0,1.0 |
| Inotropes administered **^a^** |  |  |  |
| No | 0 | 4 (31) | 4 (11) |
| Yes | 48 (100) | 9 (69) | 31 (89) |
| Summary statistics ^b^ | 0.4, 1.0,1.0,1.0,1.0 | 0.0,0.0,0.7,1.0,1.0 | 0.0,0.7,1.0,1.0,1.0 |
| Unplanned filter change on RRT whilst in PICU, n (%) |  |  |  |
| No | 48 (100) | 7 (54) | 14 (40) |
| Yes | 0 | 6 (46) | 21 (60) |

^a^ Haemodynamic status is a composite binary variable, being 1 only if either a fluid bolus or an inotrope was ever administered while on RRT.

^b^ The continuous summary statistics are the minimum, lower quartile, median, upper quartile and maximum of the proportion of days on RRT when fluid bolus/inotrope was given.

eTable 4b - Descriptive statistics of PICANet outcomes by PD, CVVH, and NIDUS (page 2)

|  | PD (n=48) | CVVH (n=13) | NIDUS (n=35) |
| --- | --- | --- | --- |
| Exposure to blood transfusion on RRT whilst in PICU, n (%) |  |  |  |
| No | 41 (85) | 1 (8) | 8 (23) |
| Yes | 7 (15) | 12 (92) | 27 (77) |
| Ventilation-free days on RRT whilst in PICU |  |  |  |
| Mean (SD) | 0.06 (0.24) | 1.54 (3.36) | 1.66 (8.32) |
| Median (IQR) | 0 (0, 0) | 0 (0, 2) | 0 (0, 0) |
| Range | 0, 1 | 0, 12 | 0, 49 |

**eTable 5 – Causes of death and relationship with devices (page 1)**

| **Index** | **Device** | **Status at 1 month** | **On renal replacement therapy at death** | **PI notes for the AE entry on MACRO reporting database** | **PI view of Causality being due to renal replacement therapy** |
| --- | --- | --- | --- | --- | --- |
| 1 | NIDUS | Alive | No | Hemophagocytic Lymphohistiocytosis type 2, leading to multi-organ failure. | No |
| 2 | NIDUS | Alive | On chronic PD | Complex unexplained illness including sepsis and hypogammaglobulinaemia, leading to multi-organ failure. | No |
| 3 | NIDUS | Dead | No | Group A streptococcal septicaemia, leading to multi-organ failure. | No |
| 4 | NIDUS | Dead | No | Hemophagocytic Lymphohistiocytosis, Veno-occlusive disease, disseminated adenovirus infection, and Stem cell transplant, leading to multi-organ failure. | No |
| 5 | Prismaflex | Alive | No | Complex congenital heart disease requiring cardiac surgery followed by cardiac arrest. | No |
| 6 | Prismaflex | Dead | No | Complex congenital heart disease requiring cardiac surgery, leading to multi-organ failure. | No |
| 7 | Prismaflex | Dead | No | Medulloblastoma treatment leading to veno-occlusive disease, leading to multi organ failure. | No |
| 8 | PD | Alive | No | Complex congenital heart disease requiring cardiac surgery, and tracheo-broncho-malacia, leading to  multi-organ failure. | No |
| 9 | PD | Dead | No | Complex congenital heart disease requiring cardiac surgery, and prematurity. | No |
| 10 | ECMO+HD | Dead | No | Complex congenital heart disease requiring ECMO, and leading to multi-organ failure. | Possibly related to ECMO complications |
| 11 | NIDUS | Dead | No | Diabetic fetopathy, hypertrophic cardiomyopathy, respiratory distress syndrome at 35 weeks gestation.  Congenital nephrotic syndrome. | No |

ECMO = Extracorporeal membrane oxygenation therapy

**eTable 5 – Causes of death and relationship with devices (page 2)**

| **Index** | **Device** | **Status at 1 month** | **On renal replacement therapy at death** | **PI notes for the AE entry on MACRO reporting database** | **PI view of Causality being due to renal replacement therapy** |
| --- | --- | --- | --- | --- | --- |
| 12 | NIDUS | Dead | No | Prematurity (32 weeks gestation), patent ductus arteriosus, necrotising enterocolitis totalis with perforation (operated), gastric necrosis and perforation (partial gastrectomy), *E Coli* sepsis. | No |
| 13 | NIDUS | Dead | No | Complex congenital heart disease requiring cardiac surgery and ECMO. *E Coli* sepsis, necrotising enterocolitis, necrotising pneumonia, pneumothoraces. | No |
| 14 | NIDUS | Dead | No | Complex congenital heart disease requiring cardiac surgery, leading to multi-organ failure. | No |
| 15 | NIDUS | Dead | No | Complex congenital heart disease requiring cardiac surgery and ECMO, and leading to multi-organ failure. | No |
| 16 | NIDUS | Dead | No | Complex congenital heart disease requiring cardiac surgery and ECMO. Cardiac arrest. | No |
| 17 | NIDUS | Dead | No | Chronic lung disease causing pneumonitis and prolonged hypoxia, leading to multi-organ failure, and pulmonary haemorrhage | SADE reported as possibly related to NIDUS therapy |
| 18 | NIDUS | Dead | No | Prematurity plus massive cystic hygroma, hypotension, and cerebral haemorrhage. Sepsis with systemic inflammatory response syndrome. | No |
| 19 | Aquarius | Dead | No | Congenital lung hypoplasia, plus congenital myopathy, requiring ECMO. | No |
| 20 | Aquarius | Dead | No | Congenital left diaphragmatic hernia (operated), hypoplastic lungs requiring ECMO, pulmonary embolism, pulmonary hypertension, bilateral chylothoraces. | No |
| 21 | Aquarius | Dead | No | Group B *Streptococcal* sepsis, persistent pulmonary hypertension, neurological injury | No |
| 22 | Aquarius | Dead | No | Congenital alveolar capillary dysplasia, treated with ECMO. | No |

ECMO = Extracorporeal membrane oxygenation therapy.

SADE = Severe adverse device event.

**eTable 6a - Non-serious adverse events (AEs) which were not consistent with the usual clinical pattern for participants requiring RRT in PICU**

| **Index** | **Device** | **Days from RRT initiation to AE start** | **Days from RRT initiation to AE resolution** | **Adverse event** | **Description** | **Causality** | **Severity** | **Action(s) taken** | **Outcome** |
| --- | --- | --- | --- | --- | --- | --- | --- | --- | --- |
| 1 | PD | 2 | 3 | Peritoneal dialysis complication | PD stopped working so removed | Definitely | Mild | Treatment adjusted/ interrupted | Resolved |
| 2 | PD | 3 | 14 | Necrotising enterocolitis neonatal | Necrotising Enterocolitis |  | Moderate | Concomitant Medication | Resolved |
| 3 | PD | 7 | 55 | Chylothorax | Chylothorax | Unrelated | Moderate | Non-drug therapy given | Resolved |
| 4 | PD | 3 | 4 | Dyskinesia | Abnormal movements - repetitive gasping associated with bilateral symmetrical upper limb extensor movement and abdominal flexion. EEG not suggestive of Seizures. MRI structurally normal. | Unrelated | Mild | Concomitant Medication | Resolved |
|  |  | 11 |  | Vocal cord paralysis | Left vocal cord palsy | Unrelated | Mild | None | Ongoing |
| 5 | PD | 19 |  | Postoperative wound infection | Surgical Site Sternal Wound Infection | Unrelated | Moderate | Concomitant Medication | Ongoing |
| 6 | PD | 18 | 49 | Chylothorax | Chylothorax | Unrelated | Moderate | Concomitant Medication | Resolved |
| 7 | Prisma-flex | 2 | 5 | Manufacturing equipment issue | Filter not working | Possible | Moderate | Treatment adjusted/ interrupted | Resolved |

Footnote: 2 patients who had incidental finding of intraventricular haemorrhage are not reported in this table, as the significance of these was unknown as routine head scanning was not usually undertaken.

**eTable 6b – Adverse device events (ADEs) only collected in intervention for NIDUS)**

| **Index** | **Device** | **Days from RRT initiation to AE start** | **Days from RRT initiation to AE resolution** | **Adverse event** | **Description** | **Causality** | **Severity** | **Action(s) taken** | **Outcome** |
| --- | --- | --- | --- | --- | --- | --- | --- | --- | --- |
| 1 | NIDUS |  | 6 | Blood pressure decreased | NIDUS was restarted post a failed trial of a furosemide infusion. Patient’s mean arterial blood pressure fell from 45 to 40. An adrenaline infusion rate was increased in response, and was subsequently weaned back down to the pre-NIDUS rate. | Possible | Mild | None | Resolved |

**eTable 6c - Serious adverse events (SAEs) (page 1)**

| **Index** | **Device** | **Days from RRT initiation to AE start** | **Days from RRT initiation to AE resolution** | **Adverse event** | **Description** | **Causality** | **Severity** | **Action(s) taken** | **Outcome** |
| --- | --- | --- | --- | --- | --- | --- | --- | --- | --- |
| 1 | NIDUS | 6 | 6 | Death | Death - Patient became bradycardic and hypotensive over hours, not responding to any treatment, and not suitable for extracorporeal membrane oxygenation, and had a cardiac arrest. Patient was not on the NIDUS at the time of deterioration. | Unrelated |  | None | Fatal |
| 2 | NIDUS | 2 | 2 | Death | Death | Unrelated |  | None | Fatal |
| 3 | NIDUS | 19 | 19 | Death | Death | Unrelated | Severe | None | Fatal |
| 4 | NIDUS | 3 | 3 | Death | Death | Unrelated | Severe | None | Fatal |
| 5 | NIDUS | 11 | 11 | Death | Death | Unrelated | Severe | None | Fatal |
| 6 | NIDUS | 22 | 22 | Death | Death | Unrelated |  | None | Resolved |
| 7 | NIDUS | 2 | 2 | Bradycardia | Bradycardia requiring chest compressions. | Unrelated | Severe | Concomitant Medication | Resolved |
|  |  | 18 | 18 | Death | Death during follow up period, following redirection of care | Unrelated | Severe | None | Fatal |
| 8 | Aquarius | 4 | 4 | Death | Death | Unrelated |  | None | Fatal |
| 9 | Aquarius | 20 | 20 | Death | Death - Patient progressively deteriorated. Care was withdrawn because his bowel was not viable. | Unrelated |  | None | Fatal |
| 10 | Aquarius | 1 | 4 | Death | Death - Group B Streptococcal Sepsis, Persistent Pulmonary Hypertension of the newborn, catastrophic neurological injury. | Unrelated | Severe | Treatment discontinued | Fatal |

**eTable 6c - Serious adverse events (SAEs) (page 2)**

| **Index** | **Device** | **Days from RRT initiation to AE start** | **Days from RRT initiation to AE resolution** | **Adverse event** | **Description** | **Causality** | **Severity** | **Action(s) taken** | **Outcome** |
| --- | --- | --- | --- | --- | --- | --- | --- | --- | --- |
| 11 | Aquarius | 19 | 19 | Death | Death – Palliation | Unrelated | Severe | None | Fatal |
| 12 | PD | 13 | 22 | Mediastinitis | Mediastinitis | Unrelated | Moderate | Hospitalisation | Resolved |
| 13 | PD | 5 | 12 | Debridement | Surgical wound debridement | Unrelated |  | Concomitant Medication | Resolved |
|  |  | 1 | 1 | Pneumothorax | Right pneumothorax, left sided white-out. Endotracheal tube pulled back. Right pleural and mediastinal drains unblocked by manipulation, with subsequent partial resolution of pneumothorax and re-expansion of left lung. | Unrelated |  | Non-drug therapy given | Resolved |
| 14 | PD | 2 | 2 | Cardiac arrest | Cardiac arrest | Unrelated | Severe | Concomitant Medication | Resolved |
| 15 | PD | 7 | 7 | Postoperative wound infection | Chest re-exploration for sternotomy wound site infection | Unrelated | Moderate |  | Resolved |

**eTable 6d - Serious adverse device event (SADE)**

| **Index** | **Device** | **Days from RRT initiation to AE start** | **Days from RRT initiation to AE resolution** | **Adverse event** | **Description** | **Causality** | **Severity** | **Action(s) taken** | **Outcome** |
| --- | --- | --- | --- | --- | --- | --- | --- | --- | --- |
| 1 | NIDUS | 2 | 2 | Pulmonary haemorrhage | Pulmonary haemorrhage in context of multi-organ failure and pulmonary hypertension, leading to death. | Possible | Severe |  | Fatal |
